# Plain packaging on tobacco products in France: Effectiveness on smokers’ attitudes one year after implementation

**DOI:** 10.18332/tid/146600

**Published:** 2022-04-07

**Authors:** Anne Pasquereau, Romain Guignard, Raphaël Andler, Karine Gallopel-Morvan, Viêt Nguyen-Thanh

**Affiliations:** 1 Santé Publique France, the National Public Health Agency, Paris, France; 2 School of Public Health, École des Hautes Études en Santé Publique, Rennes, France

**Keywords:** tobacco, plain packaging, motivation to quit

## Abstract

**INTRODUCTION:**

New packaging of tobacco products, with plain packaging and new enlarged health warnings, was made compulsory in France in 2017. This study aims to measure the impact of new packaging on smokers’ embarrassment and their motivation to quit smoking.

**METHODS:**

Data from *Santé publique France* 2016, 2017 and 2018 Health Barometer surveys were used. These randomized surveys were conducted by telephone with samples of 15216 (2016), 25319 (2017), and 9074 (2018) people aged 18–75 years. The association between smokers’ embarrassment and the influence of new packaging on motivation to quit smoking was studied using multivariate logistic regressions.

**RESULTS:**

After the introduction of new plain packaging, the proportion of smokers who felt embarrassed taking out their pack of cigarettes in plain sight because of its appearance doubled in 2017 (11.9%, 95% CI: 10.2–13.9 vs 5.9%, 95% CI: 4.4–7.8 in 2016, p<0.001) and continued to increase in 2018 (15.5%, 95% CI: 13.7–17.5, p<0.01). In 2018, women were more embarrassed than men (OR=2.0; 95% CI: 1.5–2.6, p<0.001). In 2018, 26.8% (95% CI: 24.6–29.1) of smokers said the appearance of a pack of cigarettes motivated them to quit, and 22.5% (95% CI: 18.3–27.2) ex-smokers cited it as having motivated them to quit. Smokers who were embarrassed by displaying their pack were more likely to be motivated to quit because of the pack’s appearance. People with higher incomes were less likely to report motivation to quit due to the pack than people with the lowest income (OR=0.5; 95% CI: 0.3–0.7, p<0.001).

**CONCLUSIONS:**

In the French context, the new plain packaging of tobacco products probably had an impact on smokers’ perception of tobacco by increasing the embarrassment they felt when they took out their pack of cigarettes in plain sight. It also influenced the motivation to quit smoking, and more generally, it could contribute to the denormalization of tobacco.

## INTRODUCTION

Plain (or standardized) packaging of tobacco products with large health and graphic warnings is one of the measures recommended in Article 11 of the World Health Organization’s (WHO) Framework Convention on Tobacco Control (FCTC) which focuses on the packaging and labeling of tobacco products^1,2^. In 2017, the WHO published a guide to help countries implement this recommendation and highlighted the objectives of plain packaging^3^; it is one of a set of measures intended to reduce demand, especially among young people. Australia was the first country to implement it in December 2012, followed by France in January 2017, and the United Kingdom in May 2017 (full implementation). To date, more than 20 countries worldwide have already adopted or are about to adopt plain packaging regulation (Norway, Hungary, Canada, New Zealand, Mauritius, etc.)^4^.

A Cochrane review published in 2017 concluded that ‘the available evidence suggests that standardized packaging may reduce smoking prevalence’^5^. At the time of the review only one country had implemented plain packaging, but many experimental studies suggest the expected impact. The effect of plain packaging on smoking behaviors may be explained by different processes. The first occurs through its impact on smokers’ perceptions of tobacco. Specifically, cigarettes in plain packaging are generally perceived to taste worse and be of lower quality than cigarettes in branded packaging^6^. For the youngest, the plain pack becomes less attractive and avoids the marketing strategies of the tobacco industry^7^. The second process is visual. Specifically, plain packaging strengthens the impact of health warnings as their visibility is increased and smokers perceive tobacco to be a greater risk for their health^5-7^.

As well as the above-referenced studies, which for the most part were conducted before the implementation of plain packaging, the measure’s effectiveness has also been evaluated in some of the countries where it has already been adopted. In Australia, quit attempts increased by 5.4 points one year after the introduction of plain packaging and enlarged warnings^8^. It also seemed to have an impact on smoking prevalence in the country: a drop of 0.5 points being observed after adjusting for confounding factors^5,9,10^. Furthermore, the number of smoking quitline calls increased by 78% after its introduction, and this increased activity continued over the long-term^11^. Finally, in 2017, five years after its implementation, plain packaging still had an impact (less positive and more negative perception) on Australian adolescents’ perception of cigarette packs^12^.

In the UK, MacGregor et al.^13^ pointed out that plain packaging combined with new, larger, graphic health warnings reduced the perceived attractiveness of cigarette packs among minors who smoked or were at a high risk of becoming smokers. Hiscock et al.^14^ showed that the introduction of plain packaging in the UK combined with a minimum excise duty was associated with a significant decline in sales and in tobacco industry revenues.

In France, a study conducted one year after the implementation of plain packaging with new enlarged health warnings revealed an increase in negative perceptions of tobacco products (fear of the consequences of smoking) and a decrease in smoking initiation among adolescents^15^.

The present study aims to complement the above-mentioned studies by evaluating the impact of new plain packaging on adult smokers in France. As part of the French National Tobacco Reduction 2014–2019 program (*Programme national de réduction du tabagisme,* PNRT), plain packaging was made mandatory for manufactured cigarettes and roll-your-own tobacco in 2017 (not for cigars, cigarillos, pipe or shisha). Since then, packs have been characterized by a combination of plain packaging, with only one text font and a dark green color (the same color used in Australia, the UK, etc.), and new, larger and graphic health warning content, in line with the 2014 EU Directive^16,17^.

Our study provides the first elements for an evaluation of new plain packaging (defined in this study as plain packaging with new, larger and graphic health warning content) in France in the general adult population, and focuses on three objectives:

1. Analyze whether new plain packaging increased smokers’ embarrassment when displaying their pack of cigarettes. This is a valuable indicator, since it can impact the visibility of cigarette packs in the environment, and therefore influence the perception of what is considered the norm^18^.
2. Evaluate the effect of: a) the embarrassment smokers feel when displaying their pack of cigarettes, and b) the use of an object to cover their cigarette pack, on the motivation of smokers and ex-smokers to quit smoking.
3. As previous studies have shown that the impact of anti-smoking measures differs according to individual profiles^19,20^, analyze the effect of new plain packaging according to smoker characteristics.

## METHODS

### Data

Data come from the French national Health Barometer, national representative phone surveys on random samples on the general population (aged 18–75 years) in metropolitan France, which are conducted annually by *Santé publique France* (the national public health agency). These surveys have made it possible to monitor changes in the public’s knowledge, attitudes and behaviors in terms of health since 1992. The methodology and questionnaires for these surveys are presented in the Supplementary file and in specific publications^21-23^. Questions specifically regarding plain packaging were included in the 2016, 2017 and 2018 surveys.

### Measures

Sociodemographic data collected in the three surveys included sex, age, education level (<high school diploma/high school diploma />high school diploma), occupational status (working, unemployed, student or inactive), and household income per consumption unit, first tercile for the third of the population with the lowest income, second tercile, and third tercile for the third of the population with the highest income. Consumption units are used to compare households of both different sizes and compositions by assigning a coefficient to each member of the household.

Smoking status was obtained with several questions (Supplementary file). A daily smoker was defined as an individual who either declared smoking cigarettes every day or declared the number of cigarettes (manufactured or rolled) a day. An occasional smoker was defined as an individual who declared they smoked cigarettes but not every day. In the present text, the term ‘smoker’ refers to an individual who declared they smoked cigarettes (manufactured or rolled), irrespective of whether their consumption was daily or occasional. A person who previously smoked, either occasionally or daily, and who declared they no longer smoked at the time of the survey, was considered an ‘ex-smoker’. People who reported smoking only once or twice just to try it were considered to have never smoked. Exclusive smokers of cigars, cigarillos, pipes or shisha were excluded from the analyses, not being impacted by the new plain packaging.

With quit attempts in the previous 12 months information among daily smokers, a detailed smoking status was recoded into the following classification: 1) occasional smoker, 2) daily smoker who did not try to quit during the previous 12 months, and 3) daily smoker who tried to quit during the previous 12 months.

Smokers of representative sub-samples were asked the following questions about tobacco packaging: 1) ‘Do you ever feel embarrassed by displaying your pack of cigarettes because of its appearance?’ (systematically/often/sometimes/rarely/never). For the analyses, responses were grouped together as follows: ‘yes’ grouped ‘systematically’ and ‘often’; while ‘no’ grouped ‘sometimes’, ‘rarely’ and ‘never’; and 2) ‘During the past twelve months, have you used a cigarette case, box, cover or pouch for your cigarettes to hide warnings or photos?’ (yes/no).

To measure the effect of the appearance on the motivation to quit, smokers and ex-smokers were asked: ‘Does/Did the appearance of the packs of cigarettes or tobacco motivate you to stop smoking?’ (absolutely/quite a lot/ not really/ not at all). For the analyses, responses were grouped together as follows: ‘yes’ grouped ‘absolutely’ and ‘quite a lot’; while ‘no’ grouped ‘not really’ and ‘not at all’.

Table 1 details the sub-samples and questions related to plain packaging in the 2016, 2017 and 2018 Health Barometer surveys concerned.

**Table 1:** Sub-samples of responding smokers and ex-smokers and questions related to plain packaging in the 2016, 2017 and 2018 Health Barometer surveys

*Questions*	*Population*	*2016 (N=15216) n*	*2017 (N=25319) n*	*2018 (N=9074) n*
Do you ever feel embarrassed by displaying your pack of cigarettes in plain sight because of its appearance?	Smokers	1465	1728	2339
During the past twelve months, have you used a cigarette case, box, cover or pouch for your cigarettes to hide warnings or photos?	Smokers			2391
Does/did the appearance of the packs of cigarettes or tobacco motivate you to stop smoking?	Smokers Ex-smokers			2392 596

### Analyses

- The changes over time in embarrassment taking out a pack of cigarettes or tobacco in plain sight between 2016 (before implementation), 2017 (a few months after) and 2018 (one year later) were statistically tested using Pearson’s χ^2^ test with a Rao-Scott second order correction to take into account the sampling plan.
- Factors associated with embarrassment in 2018 were analyzed using a logistic regression. The following variables were included: sex, age, income, education level, occupational status, smoking status and the number of cigarettes smoked per day.
- The second part of the results section presents the proportion of smokers who declared they were motivated to quit by the appearance of the package.
- Logistic regressions were performed to study the links in 2018 between the motivation to quit because of the appearance of the pack (dependent variable) and: a) the use of an object to cover their pack (explanatory variable), and b) the embarrassment displaying their pack of cigarettes or tobacco (explanatory variable). Both regressions were adjusted for the following variables: sex, age, income, education level, occupational status, smoking status, and the number of cigarettes smoked per day. The use of an object to cover the pack was added as an adjustment variable in the second model. The interactions between embarrassment taking out a pack of cigarettes or tobacco in plain sight and each of the other variables of the model were tested using an interaction term (embarrassment–age for example). The model was stratified in case of significant interaction at the 5% threshold level.

## RESULTS

### Embarrassment taking out cigarette pack in plain sight because of its appearance

*Evolution between 2016 (i.e. before the implementation of plain packaging), 2017 and 2018*

In 2016, before the introduction of plain packaging with new enlarged health warnings, overall, 5.9% (95% CI: 4.4–7.8) of smokers were embarrassed by displaying their pack of cigarettes because of its appearance (Table 2).

**Table 2:** Responses to the question ‘Do you ever feel embarrassed displaying your pack of cigarettes or tobacco because of its appearance?’ and changes over the 2016–2018 period

*Responses*	*2016 Before implementation %*	*2017 A few months after %*	*2016–2017 p*	*2018 One year later %*	*2017–2018 p*	*2016–2018 p*
**Yes**	**5.9**	**11.9**	**<0.001**	**15.5**	**<0.01**	**<0.001**
Systematically	1.7	6.0		6.4		
Often	4.2	5.9		9.1		
**No**	**94.1**	**88.1**		**84.5**		
Sometimes	6.8	10.1		10.6		
Rarely	8.2	8.3		10.3		
Never	79.2	69.7		63.6		

Source: *Santé publique France* Health Barometer surveys 2016, 2017 and 2018.

In 2017, a few months after the introduction of new plain packaging in France, the proportion of smokers who were embarrassed by displaying their pack because of its appearance doubled (11.9%, 95% CI: 10.2–13.9). Conversely, the proportion of smokers not embarrassed decreased from 94.1% (95% CI: 92.2–95.6) to 88.1% (95% CI: 86.1–89.8).

In 2018, more than a year after the introduction of new plain packaging, embarrassment continued to increase, with 15.5% (95% CI: 13.7–17.5) of smokers feeling embarrassed. The proportion of smokers not embarrassed continued to decrease (from 88.1%, 95% CI: 86.1–89.8 to 84.5%, 95% CI: 82.5–86.3).

*Factors associated with embarrassment by displaying pack in 2018*

In 2018, after the introduction of new plain packaging in France, the embarrassment felt by smokers displaying their pack of cigarettes was associated with age, in the adjusted model: for each age group between 25–75 years, smokers were more likely to be embarrassed than people aged 18–24 years (Table 3). Women were more embarrassed than men (OR=2.0; 95% CI: 1.5–2.6, p<0.001). Income was also associated with embarrassment: people with the higher incomes were less likely to feel embarrassed (OR=0.6; 95% CI: 0.4–0.9, p<0.05 for the 2nd and 3rd terciles) than people with the lowest income (1st tercile). Furthermore, students, retirees and other inactive people were less embarrassed than employed workers (OR=0.6; 95% CI: 0.4–0.9, p<0.05).

**Table 3:** Factors associated with being embarrassed (systematically or often) to take out a pack of cigarettes in plain sight because of its appearance in 2018 (N=2319)

*Factors*	*n*	*%^a^*	*OR*	*95 % CI*
**Sex**		***		
Men (Ref.)	1207	11.6	1	
Women	1132	19.9	2.0***	1.5–2.6
**Age** (years)		**		
18–24 (Ref.)	303	6.9	1	
25–34	437	15.7	2.2*	1.1–4.2
35–44	535	19.8	2.8**	1.4–5.3
45–54	521	16.4	2.1*	1.1–4.1
55–75	543	16.2	2.5**	1.3–4.8
**Income per consumption unit**		*		
1st tercile (low) (Ref.)	701	19.4	1	
2nd tercile	781	13.7	0.6*	0.4–0.9
3rd tercile (high)	653	12.7	0.6*	0.4–0.9
Don’t know/refused to answer	204	13.2	0.7	0.4–1.3
**Educational level**		*		
<High school diploma (Ref.)	970	17.7	1	
High school diploma	549	12.1	0.7	0.5–1.1
>High school diploma	813	14.1	0.8	0.5–1.1
**Occupational status**				
Employed (Ref.)	1565	15.8	1	
Unemployed	220	20.3	1.2	0.7–1.9
Student, retirees, other inactive status	554	12.2	0.6*	0.4–0.9
**Smoking status**				
Daily smoker with no quit attempt in previous 12 months (Ref.)	1456	17.0	1	
Daily smoker with quit attempt in previous 12 months	470	11.9	0.8	0.5–1.1
Occasional smoker	412	14.5	0.7	0.4–1.1
**Number of cigarettes smoked daily**				
0–5 (Ref.)	762	18.0	1	
5–10	672	13.8	0.5**	0.3–0.8
>10	892	15.0	0.6*	0.4–0.9

* p<0.05,

** p<0.01,

*** p<0.001.

a Percentages of smokers who felt embarrassed, and p for bivariate analyses between individual characteristics variables and embarrassment.

Source: *Santé publique France* Health Barometer surveys 2016, 2017 and 2018.

The number of cigarettes smoked per day was associated with smokers’ embarrassment displaying their pack of cigarettes: those who smoked 5–10 cigarettes per day (OR=0.5; 95% CI: 0.3–0.8, p<0.01) and those who smoked ≥10 cigarettes per day (OR=0.6; 95% CI: 0.4–0.9, p<0.05) were less embarrassed than those who smoked <5 cigarettes per day.

### The effect of new plain packaging on motivation to quit smoking

*Motivation to quit smoking*

In 2018, 22.5% (95% CI: 18.3–27.2) of ex-smokers said the appearance of a pack of cigarettes had motivated them to quit. For ex-smokers, the multivariate analysis showed no association between motivation because of the pack’s appearance and the various sociodemographic variables studied (sex, age, income, educational level, occupational status). Among smokers in 2018, 26.8% (95% CI: 24.6–29.1) said the appearance of a pack of cigarettes motivated them to quit. Conversely, 73.2% (95% CI: 70.9–75.4) said it was not a motivating factor.

*Use of an object to hide their cigarette pack*

In 2018, 17.2% (95% CI: 15.3–19.1%) of smokers said they used an object (i.e. case, box, cover or pouch) for their cigarette pack to hide warnings and photos. In the adjusted model, only sex was linked to this avoidance strategy, with women being more likely to use a cover than men (OR=2.4; 95% CI: 1.8–3.2, p<0.001).

*Association between embarrassment, the use of an object to hide cigarette pack, and motivation to quit smoking*

Embarrassment taking out a pack of cigarettes in plain sight because of its appearance was associated with motivation to quit smoking. Among embarrassed smokers, 51.3% (95% CI: 44.6–57.9) were motivated to quit by the pack’s appearance. Conversely, only 22.2% (95% CI: 20.0–24.5) of smokers who reported not being embarrassed reported that the appearance of the pack motivated them to quit.

The use of an object (case, box, cover, pouch) to hide warnings or photos was also associated with motivation to quit. Among smokers using such an object, 35.5% (95% CI: 29.9–41.4) said they were motivated to quit because of the appearance of the pack. This contrasts with 25.0% (95% CI: 22.7–27.5) for those who did not use any such object.

In the multivariate logistic regression modeling, the motivation to quit smoking due to the pack appearance, without the explanatory variable ‘embarrassment taking out a pack of cigarettes or tobacco in plain sight’, and controlling for socioeconomic and tobacco consumption characteristics, the use of an object to hide the pack was associated with the motivation to quit smoking (OR=1.7; 95% CI: 1.3–2.3, p<0.001).

In the adjusted model with the explanatory variable ‘embarrassment taking out a pack of cigarettes or tobacco in plain sight’ (Table 4), smokers who systematically (OR=4.6; 95% CI: 2.9–7.4, p<0.001), often (OR=5.7; 95% CI: 3.8–8.6, p<0.001), sometimes (OR=4.3; 95% CI: 2.9–6.2, p<0.001), or rarely embarrassed (OR=1.7; 95% CI: 1.1–2.5, p<0.05) all had a higher probability of being motivated to quit by the appearance of the pack than smokers who were never embarrassed. In contrast, the use of an object to hide the pack was no longer associated with motivation to quit smoking (OR=1.0; 95% CI: 0.7–1.4). The association between the use of an object to hide the pack and motivation to quit may therefore be explained by the embarrassment felt.

**Table 4:** Factors associated with motivation to quit smoking due to the appearance of packs of cigarettes or tobacco in 2018 (N=2308)

*Explanatory variables*	*n*	*%^a^*	*OR*	*95 % CI*
**Sex**		*		
Men (Ref.)	1228	24.5	1	
Women	1164	29.3	1.0	0.8–1.3
**Age** (years)				
18–24 (Ref.)	307	27.6	1	
25–34	446	24.3	0.8	0.5–1.3
35–44	542	28.5	1.0	0.6–1.6
45–54	535	27.5	1.1	0.7–1.7
55–75	562	26.4	1.1	0.7–1.8
**Income per consumption unit**		**		
1st tercile (low) (Ref.)	711	31.2	1	
2nd tercile	799	26.1	0.8	0.6–1.1
3rd tercile (high)	672	19.7	0.5***	0.3–0.7
Don’t know/refused to answer	210	29.2	0.8	0.5–1.3
**Educational level**				
<High school diploma (Ref.)	990	28.3	1	
High school diploma	553	23.7	0.8	0.6–1.1
>High school diploma	841	26.0	0.9	0.6–1.2
**Occupational status**				
Employed (Ref.)	1600	26.2	1	
Unemployed	222	27.9	0.9	0.5–1.4
Student, retirees, other inactive status	570	27.8	0.9	0.6–1.2
**Smoking status**		***		
Daily smoker with no quit attempt in previous 12 months ( Ref. )	1471	22.2	1	
Daily smoker with quit attempt in previous 12 months	473	33.2	2.0***	1.5–2.8
Occasional smoker	447	35.7	2.0**	1.3–3.0
**Number of cigarettes smoked daily**		***		
0–5 (Ref.)	803	34.4	1	
5–10	676	27.2	0.9	0.6–1.3
>10	900	20.7	0.6**	0.4–0.9
**Embarrassment taking out cigarette pack in plain sight**		***		
Never (Ref.)	1491	17.3	1	
Rarely	248	26.0	1.7*	1.1–2.5
Sometimes	252	47.5	4.3***	2.9–6.2
Often	203	53.3	5.7***	3.8–8.6
Systematically	138	48.5	4.6***	2.9–7.4
**Use of an object to hide pack**		***		
No (Ref.)	1965	25.0	1	
Yes	416	35.5	1.0	0.7–1.4

* p<0.05,

** p<0.01,

*** p<0.001.

a Percentages of smokers who declare being motivated to quit smoking due to the pack appearance, and p for bivariate analyses between individual characteristics variables and motivation.

Source: *Santé publique France* Health Barometer 2018.

*Factors associated with motivation to quit smoking*

The motivation of smokers to quit smoking generated by the appearance of the pack was not strongly associated with their socioeconomic characteristics, in the adjusted model. Specifically, only income level was associated with motivation (Table 4). People with higher incomes were less likely to report motivation to quit than people on the lowest income (OR=0.5; 95% CI: 0.3–0.7, p<0.001). Conversely, tobacco consumption characteristics were more strongly associated with motivation to quit. In terms of smoking status, daily smokers who made a quit attempt within the previous 12 months and occasional smokers were more motivated to quit by the appearance of the pack than smokers who had not made a quit attempt in the previous 12 months (OR=2.0 for the 2 groups, 95% CI: 1.5–2.8, p<0.001 and 95% CI: 1.3–3.0, p<0.01, respectively). Those who smoked ≥10 cigarettes per day were less likely to be motivated by the appearance of the package than those who smoked <5 cigarettes per day (OR=0.6; 95% CI: 0.4–0.9, p<0.01).

The study of the interactions between the embarrassment taking out a pack of cigarettes in plain sight and each other variable of the model highlighted that the embarrassment felt was associated in different ways with motivation to quit, generated by the appearance of the pack for the following two characteristics:

- Occupational status (p-interaction=0.016): among employed, retirees, students, and other inactive persons, embarrassment was associated with greater motivation (OR=5.0; 95% CI: 3.4–7.5, p<0.001 and OR=3.1; 95% CI: 1.5–6.8, p<0.01, respectively). Instead, it was not associated with motivation in unemployed people (OR=0.9; 95% CI: 0.3–2.6).
- Using an object to hide cigarette pack (p<0.001): embarrassment was associated with motivation to quit smoking only among people who did not use such an object (OR=5.7; 95% CI: 3.8–8.7, p<0.001 vs OR=1.5; 95% CI: 0.9–2.6 among those who did use such an object).

## DISCUSSION

### The effect of new plain packaging on smokers’ embarrassment

The results of the present study suggest that the new packaging of tobacco products in France has had an impact on the embarrassment smokers felt when displaying a pack of cigarettes or tobacco due to its appearance. Smokers were twice as likely to feel embarrassed following the introduction of plain packaging with new enlarged graphic health warnings in France in 2017 with respect to before its introduction in 2016. This proportion continued to increase in 2018.

The embarrassment felt by smokers is an indicator of the denormalization of tobacco. A review of the literature performed in 2018 showed that plain packaging participated in tobacco denormalization by increasing negative opinions about smoking and about starting to smoke^24^.

It also participates in the denormalization of tobacco in the overall environment (familial, social, visual, etc.). In Australia, a recent study showed that cigarette packs were much less present in the environment (outdoor seating areas, bars, restaurants) two years after the introduction of plain packaging in places where children were present^25^.

### The effect of embarrassment on motivation to quit smoking

In the present study, the appearance of a cigarette or tobacco pack was a factor that motivated more than a quarter of the study’s smokers to quit, and more than a fifth of ex-smokers cited it as having motivated them to quit. Our results show that smokers who were embarrassed to display their pack of cigarettes or tobacco because of its appearance, were more likely to be motivated to quit because of the appearance of the pack. Indeed, a review of the literature published in 2018 concluded that plain packaging was effective in increasing the intention to quit smoking among exposed individuals^24^. The impact study conducted in Australia also suggested an effect of plain packaging on smoking prevalence^9^.

However, the use of an object to hide cigarette packs may mitigate or prevent the impact of plain packaging and health warnings on motivation to quit smoking. Indeed, in the present study, embarrassment was associated with motivation to quit only in people who did not use an object to hide their cigarette pack. This result was not found among all smokers in another study in Australia which highlighted an overall positive impact on smokers, and rejection of the measure among only a minority of them^26^. We conclude that longer follow-up is necessary to see if this impact is temporary or continues over time.

Accordingly, plain packaging with new enlarged health warnings can act on the following two dimensions described in behavioral change models, in particular COM-B model: 1) motivation, an individual component; and 2) opportunity, through its social and environmental component and the implementation of a new norm^27^.

### Factors associated with embarrassment by displaying pack and motivation to quit

In the present study, women were more likely to report both embarrassment when displaying a pack of cigarettes or tobacco and to use of an object to hide packs. A Spanish study also showed that women were more sensitive than men to cigarette packaging, and more impacted by it. Furthermore, plain packaging influenced women’s perceptions more^28^. For decades, the tobacco industry has developed marketing strategies targeting women, by associating cigarettes with glamor and a positive social image^29^. Plain packaging has made some of these marketing strategies impossible, with a bigger impact on women than men.

In our study, the motivation to quit smoking brought about by new plain packaging was lower among people with the higher incomes. In France, social inequalities linked to smoking continue to be very pronounced^30^. Our results suggest that the impact of plain packaging with new enlarged graphic health warnings is possibly stronger on more socially precarious populations, and that the measure contributes towards meeting one of the primary objectives of the national program against tobacco.

### French results confirming the results of previous studies over the medium term and in the general population

The present study conducted after new plain packaging was implemented in France, confirms the results of studies on smokers’ perceptions of plain packaging conducted before its implementation. An experimental study conducted in France in 2011 showed that plain packs were perceived to be less attractive than ordinary packs, less likely to motivate young people to buy tobacco, and more likely to strengthen motivation to quit in smokers already motivated to some degree^31^. Moreover, a before/after implementation study of adolescents found greater fear of the consequences of smoking, and perception of less acceptability of smoking by family and friends^15^.

### Strengths and limitations

The present study has several limitations. First, as the data for the indicators presented here came from self-reported surveys, reporting bias cannot be ruled out. Second, the question about respondents’ perception of the package was only asked to smokers. Had it been asked to ex-smokers who had recently quit, it could have provided more specific information about this sub-population. Third, although data on the motivation to quit smoking generated by the appearance of the package were collected for ex-smokers, the number of respondents was relatively small. Furthermore, data on the characteristics of their past use were not collected. Both these elements may certainly have impacted the multivariate analysis. Fourth, a causal link cannot be established from cross-sectional surveys, associations are studied, and a possible reverse causality cannot be excluded. Finally, since plain packaging and new enlarged health warnings were implemented simultaneously, we cannot distinguish the impact of each measure separately. In Canada, plain packaging without any changes regarding the size and content of health warning labels was implemented in 2020. A study suggests that plain packaging reduces appeal but does not have an impact on health warning effectiveness if they are not renewed^32^.

The study also has several strengths. First, it is based on robust surveys, which are representative of the French general population. The large sample size made it possible to highlight social disparities.

Furthermore, the study included several measurement time points, which made it possible to assess the effects of the introduction of plain packaging both in the short and medium terms.

Finally, general population surveys with this robustness which explore the relationship of smokers with their pack of cigarettes are rare, particularly those which examine the use of an object to hide the pack.

## CONCLUSIONS

It is difficult to measure separately the effect of each and every French tobacco control measure on the decrease in the smoking prevalence since 2016^32^ because most were simultaneously implemented between 2016 and 2018 (price increases, reinforced quitting support, social marketing campaigns, plain packaging with new enlarged health warnings). Nevertheless, the new packaging of tobacco products (larger health warnings and plain packs) has probably had an impact on smokers’ behaviors in France, through the embarrassment felt when taking out a pack in plain sight, and their motivation to quit caused by the appearance of the pack. What is certain is that this measure has contributed – in a particularly strong anti-tobacco context – to further denormalize tobacco products in France. This strategy of denormalization needs to be continued in France, and our results show the value of implementing plain packaging with new enlarged graphic health warnings in countries where it has not yet been adopted.

## Data Availability

The data supporting this research are available from the authors on reasonable request.
