# Supplementary material for: Plain packaging on tobacco products in France: Effectiveness on smokers’ attitudes one year after implementation
Source: Tob Induc Dis. 2022 Apr 7;20:35. doi: 10.18332/tid/146600 (PMC8985692; doi:10.18332/tid/146600)
Supplement: Supplementary file 1 [file TID-20-35-s1.pdf]

Supplementary material: Plain (standardized) cigarette pack in France since 1 January 2017

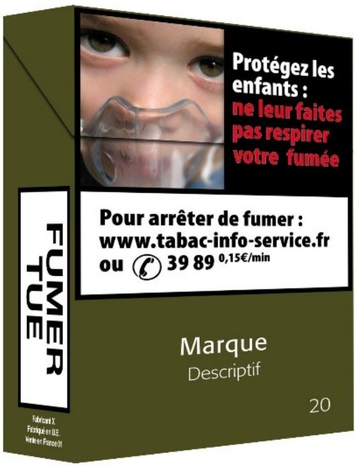

Appendix 1: Description of the French national Health Barometer surveys

Data collection

Health Barometers are annual cross-sectional French national representative surveys on random samples of general population set up by Santé publique France. They are conducted by telephone by the Ipsos survey firm, based on a two-stage random sampling design, specifically, first the household and then one person in the household. Data are weighted to take into account the probability of inclusion, then adjusted for the population residing in metropolitan France according to sex as a function of age, region of residence, size of urban unit, level of education, and number of people living in the household.

The main themes addressed in the 2016 survey were vaccination and sexual health. The 2017 edition collected data on substance use (tobacco, alcohol, illicit drugs), mental health, physical activity, and vaccination. In 2018, the survey was exclusively dedicated to the theme of tobacco smoking. In 2016 and 2017, some parts of the questionnaires were asked to randomly selected subsamples of the whole sample, in order to reduce the length of the interviews and thus to favor participation rates.

The 2016, 2017 and 2018 surveys were conducted on respectively 15,216 people aged 15-75 years old, 25,319 people aged 18-75 years old and 9,074 people aged 18-75 years old. The participation rates were respectively 50.0%, 48.5% and 55.6%. The analyzes focused on the 18-75 age group, population common to the three surveys. Questions about tobacco packaging were asked only among smokers of manufactured cigarettes and roll-your-own tobacco: subsample of smokers in 2016 (N=1,465); subsample of smokers in 2017 (N=1,728); the whole of smokers and ex-smokers in 2018 (N=2,339 and N=596 respectively).

Smoking status

Smoking status at the time of the questionnaire was obtained with three questions: “Do you smoke tobacco, even if only occasionally?” Those who answered “yes” to the first question were then asked: “Do you smoke every day?” and “How many cigarettes (manufactured or roll-your-own) do you smoke on average? (per day, week, month or year)”. Smokers reporting smoking every day (second question) or reporting a certain number of cigarettes per day (third question) were considered as daily smokers. All other smokers were considered as occasional smokers.

Non-smokers (i.e. those replying ‘no’ to the first question) were asked: ‘In your lifetime, have you ever tried smoking tobacco?’ and if they answered ‘yes’, they were then asked, ‘Did you smoke just once or twice, just to try it, or occasionally or daily for less than 6 months, or daily for more than 6 months?’.

Quit attempts in the previous 12 months were measured only among daily smokers with the question “Have you ever voluntarily quit smoking for at least a week?”. Those who replied “Yes” were then asked “When was the last time you tried to quit smoking?”.

© 2022 Pasquereau A. et al.
